# A New Method of Amputation

**Published:** 1858-12

**Authors:** 


### ECLECTIC DEPARTMENT,

### AND SPIRIT OF THE MEDICAL PERIODICAL PRESS.

---

### *A New Method of Amputation.* By M. Maisonneuve.

M. Maisonneuve read before the Académie des Sciences, April 26th, 1858, a note on a new operation for amputation, which he calls the *diaclastic method.* The peculiarity of this method is, that for its execution neither the knife is used for dividing the muscles, nor the saw for cutting the bones, nor permanent ligatures to arrest hæmorrhage; and that, contrary to the ordinary methods, the division of the bone constitutes the first step of the operation, and precedes the division of the soft parts.

The principal object of this method is to avoid the occurrence of purulent infection, by substituting for the ordinary process of division by cutting instruments, the process of breaking, tearing, and extemporaneous ligature, the contusing action of which obliterates effectually the vascular orifices.

M. Maisonneuve uses for the execution of this method an *osteoclast,* or instrument for breaking the bone; and a powerful *serre-nœud* for the division of the soft parts. The author describes the operation in the following manner:

"The patient having been brought under the influence of chloroform, the surgeon applies the osteoclast precisely on the spot where he intends to break the bone, taking care to protect the soft parts in contact with the instrument by thick compresses; then, giving the screw several turns, he produces the fracture; he removes the instrument, and immediately substitutes the serre-nœud, in the metallic loop of which he embraces the member ten or fifteen centimetres below the point of fracture; then, turning the screw, he gradually compresses the tissues until the circulation in the vessels is interrupted. This done, he divides the muscles to the bone by a circular incision with the bistoury, two or three centimetres below the serre-nœud, tears off by a twisting movement the extremity of the member which is attached merely by same adhering portions of muscular tissue, and finishes the operation by continuing to turn the screw of the serre-nœud until the tissues embraced in the loop of the ligature are completely divided. If the last step of the operation is conducted with prudent slowness, not a drop of blood will ooze from the wound resulting from the amputation, whatever the amputated member may be."

This singular method has been applied with success to five amputations of the leg and to one of the forearm.—*Archives Générales,* June, 1858, from *Medico-Chirurgical Review.*

*Rationale of the Saccharine Treatment of Diabetes.*—Dr. JOHN SLOANE, in a paper read before the Leicester Medical Society (April 20, 1858), gives the following rationale of the saccharine treatment of diabetes:

"Glucose, the variety of sugar found in the urine of diabetics, is generated in the livers of animals throughout the animal kingdom, almost wholly irrespective of the nature of their food. The glucose secreted by the hepatic cells passes into the hepatic veins, thence into the inferior vena cava, and th'ough the right side of the heart to the lungs, where, being exposed to the atmosphere, it sometimes completely disappears. Mr. Bernard has found sugar in the livers of mammals, of birds, of reptiles, of fishes, of molluscs, and of articulated animals. He has found it in omniverous, herbivorous, and carniverous animals. That the secretion of sugar is independent of the nature of the food, he proves by many experiments, of which I shall mention the following. He fed dogs exclusively on flesh for six or eight months; and when they were killed at the expiration of that period, he found as much sugar in their livers as in those of dogs fed upon upon a mixed diet. Owlets taken in their nests were fed exclusively on raw bullock's liver for three months, and were then killed; their livers always contained the normal quantity of sugar. Two dogs were fed solely on flesh, and two on amylaceous and saccharine food; they were all killed at as nearly as possible the same period of digestion, and the results of the chemical examination of their livers showed that the quantity of sugar secreted did not depend on the nature of their diet.

"Rollo recommended the use of fat for diabetics. M. Thenard and Dupuytren made them eat lard. We have fed dogs with lard and axunge; and we have fouud this very curious fact, that, under the influence of this alimentation, the sugar diminished in the liver absolutely in the same manner as if the animal had been kept fasting. In dogs to which M. Bernard has given nothing but pure water, he has found the secretion of sugar kept diminishing, and it ceased to appear about three or four days before its death. For the first thirty-six hours, the quantity continues considerable, but during the following days it diminishes very rapidly.

"A dog, having fasted thirty-six hours, had a copious repast of boiled sheep's head, and, three hours afterwards, was killed. The blood in the portal vein, previous to its entrance into the liver, contained no trace of sugar; whereas, in the blood from the hepatic veins, there was a considerable quantity. This experiment, writes Bernard, would alone suffice to cause one to admit, as a natural and necessary conclusion, that the sugar is produced in the liver; yet we have accumulated proofs of every kind about this proposition; and we have shown that the hepatic tissue constantly contained sugar, and that it was the only tissue of the body which offered this character.

"In animals fasting, the blood which arrives at the liver presents no trace of sugar; that which leaves it contains a cousiderable quantity. Inversely, the blood which arrives in the lungs contains sugar; and that which leaves it presents no trace of this substance. The sugar in this physiological state remains hidden between the liver and the lung, and does not show itself at the exterior. This statement is true only in an animal fasting. When the digestion commences, the quantity of sugar gradually augments; yet during the two or three hours following the ingestion of aliment, notwithstanding the increase of the saccharine secretion, all the sugar can be destroyed before it arrives at the arterial system; and it is only after the lapse of time that the production of sugar, surpassing the limits of destruction, becomes temporarily excessive in the organism. At this period of digestion, one finds sugar in all the vessels of the body, arterial and venous, and even in the renal arteries; but the proportion is too slight for any of the sugar to pass in the urine. Yet we shall see that, under certain physiological circumstances, the quantity of sugar can be increased to the point that it passes off in the urine without the animal being diabetic. Under the ordinary circumstances of digestion, this species of saccharine overflowing is manifested equally with animal or amylaceous diet, and it lasts about three or four hours. It is not less than six or seven hours after a meal that the excess of sugar in the blood commences to disappear, and that the equilibrium between its production and its destruction tends to re-establish itself as before digestion. This species of oscillation, which the glycogenic function presents, it is very important to know; for in the pathological state (diabetes) we find exactly the same phases, with the exaggeration we should expect in this malady. Different observers—Rayer in France, and Traube in Germany—have remarked that there are diabetics which do not pass sugar in their urine, except at the time of their digestion; and that, in the interval, their urine does not contain sugar. This phenomenon can be reconciled very naturally with the physiological fact which has been pointed out to you. There is nothing essentially different between the normal state and the pathological symptom, save the intensity of the phenomenon caused by a deviation of vital activity.

"The sugar is formed from the albuminous substances; and this sugar is the result of the physiological action of the liver upon those principles, which are divided so that their oxygen, hydrogen, and carbon, are grouped so as to form sugar, whilst their azote enters into other combinations, and probably into the azotized principles of the bile. One does not know, indeed, any other origin for the saccharine matter, which cannot be produced in the intestine without digestion. Experiment has shown us that, during alimentation by means of albuminous substances, the intestine and the blood of the portal vein never contain saccharine matter of any kind. Neither gelatine or flesh produce saccharine matter in the intestinal tube by the known digestive processes. The amylaceous matters taken as food enter as sugar into the portal vein, and, arriving at the liver in this state, are then destroyed by this organ and changed into an emulsion *par une matière protéique spéciale*. We have said that the sugar introduced into the intestinal tube does not augment the quantity of this matter contained in the liver, but that it is there destroyed, and causes the appearance of an emulsive substance. That the sugar introduced into the intestinal canal does not augment the quantity of this matter contained in the liver, M. Bernard shows by the following experiments. He takes two rabbits, whose urine he first finds, by testing, to be free from sugar. Into the stomach of one he injects a quantity of sugar in solution, with some ferrocyanide of potassium. Beneath the cellular tissue he injects half the quantity of an exactly similar solution. He examined their urine an hour afterwards, and he finds in that of the first not the least trace of sugar, while the urine of the second presents it in considerable quantities. But you may say that this difference may be accounted for by the intestinal absorption being less rapid than the subcutaneous; but in both the ferrocyanide of potassium was readily detected in the urine. This will prove that the absorption is equally effectual in the intestine as under the skin, but that, in the first case, the solution has abandoned one of its constituents, the sugar, in traversing the liver; whereas this has not taken place in the second instance. He arrives at similar results in the following experiments. Through a small opening in the abdomen of a rabbit, he injects a quantity of the same solution into one of the branches of the portal vein; and into the jugular vein of another rabbit he injects the same quantity of the same solution. It is clear that, in this mode of operating, we cannot have any difference in the absorption, as in both cases we introduce the substances directly into the blood. Nevertheless, we obtain exactly the same result; that is to say, that in the rabbit, in which we injected by the jugular, the sugar has passed into the urine with the ferrocyanide of potassium, and with very great rapidity; whilst in the rabbit injected by the portal vein, the ferrocyanide of potassium alone will have passed into the urine, where one cannot find the least trace of sugar. These experiments are very conclusive. Bernard proves by experiment that starch, taken as food in the intestine by the influence of the pancreatic juice, becomes converted into sugar; and this passes into the portal vein. That sugar is destroyed by the liver, receives further confirmation, he states, by the facts known in the fattening of cattle. You all know that animals fatten most by the use of food in which starch predominates; that the geese and the ducks, in which the fat livers are artificially produced, are gorged with a *paté* of maize or other amylaceous food: that the fat formed by an animal is not in proportion with the adipose matter which it takes; that, on the contrary, the animals which only eat fat, far from becoming fat, get lean rapidly. Hereafter it is not only the biliary secretion which we shall have to look upon in the liver; it has two other functions of capital importance—one the production of sugar, which is dependent upon the aliment containing albuminous matters; the other, the production of fat, which is dependent upon the amylaceous and saccharine matters in the food.

"Cane-sugar is never destroyed; it is constantly eliminated by the urine when it is injected directly into the blood; but this sugar, when in the intestine, is in part, at least, transformed into glucose. The latter, on the contrary, injected into the blood, can be destroyed in certain proportions.

"When we prick the mesial line of the floor of the fourth ventricle, in the exact centre of the space between the origins of the auditory and pneumogastric nerves, we produce an exaggeration of the hepatic (saccharine) function, and of the renal secretion; if the puncture be effected a little higher, we very often only produce an augmentation in the quantity of the urine, which then frequently becomes charged with albuminous matters; while, if the puncture be below the indicated point, the discharge of sugar alone is observed, and the urine remains turbid and scanty. Hence it appears that we may distinguish two points, of which the inferior corresponds to the secretion of the liver, and the superior to that of the kidneys. As, however, these two points are very near to one another, it often happens that, if the instrument enters obliquely, they are simultaneously wounded; and the animal's urine not only becomes superabundant, but at the same time saccharine. The urine becomes saccharine in from one to two hours after the operation, but seldom continues for more than a day.

"The secretion of sugar is not under the direct influence of the pneumogastric nerve; for if it be divided before irritating the floor of the fourth ventricle, sugar still appears in the urine. Bernard believes that the influence is transmitted by reflex action through the ganglia of the sympathetic.

"There is a phenomenon which is manifested, for example, when, after fasting a certain time, a great quantity of sugar is taken. The intestinal absorption then proceeds with extreme rapidity. A great quantity of sugar arrives in mass in the liver; the mechanical circulation much prevails over the chemical; the sugar is poured into the general circulation in proportion much greater than occurs in the normal state; and it passes then into the urine, where its short-lived presence can be found for a certain time.

"M. Bernard, after a great many experiments in reference to the subject, has proved that there is a species of election in the excretion of matters which pass out of the organism. Sugar is eliminated in two ways only—by the kidneys, and by the mucous membrane of the stomach. When sugar is ejected into the blood of an animal to saturation, and puts it for a time into a state of diabetes, we do not find sugar in the saliva, in the tears, pancreatic juice, bile, nor perspiration: whilst the urine and gastric juice contain it in proportions more or less notable. These results entirely resemble those obtained in diabetic patients. Lehmann states, however, that he has obtained sugar from the saliva of a diabetic. The presence of sugar has been pointed out in the expectoration of diabetics. Bernard admits that sugar can be had in a notable quantity in the expectoration. But, he writes, we must not confound the bronchial mucus which these patients, almost always phthisical, in the last stage of the disease expel in abundance, with the salivary secretion properly so called; it is the mucosities formed in the lung which contain the saccharine matter. Nevertheless, this fact is not constant; for M. Raynor has reported to the Society of Biology a case in which the expectoration of a phthisical patient examined by M. Wurtz did not contain sugar. Bernard proves by the following experiments the statements regarding the election in excretion of matters which pass out of the organism.

"He takes a dog with a parotidean opening, into which he inserts a tube. Nothing flows by this tube, which proves that the secretion is not continuous. By putting in the mouth some vinegar he excites the flow of saliva, which passes out of the tube rapidly in large drops. He next injects into the jugular vein of the animal a solution containing sugar, prussiate of potash, and iodide of potassium. Immediately after this injection the salivary secretion is again excited in the same way. The saliva is received into three glasses. One is examined for sugar, and none is found. The sugar, therefore, does not pass in the saliva. The second is examined for prussiate of potash, and it is not present. The third is found to contain iodide of potassium. This substance then passes immediately into the saliva, whilst the prussiate of potash and the glucose, equally soluble, cannot be found. In the saliva extracted before the injection, none of the substances exist. In the urine of the same animal, after the injection. the prussiate of potash is found in considerable quantity, and the iodide of potassium in small proportion. As regards the sugar, there is none yet, but we shall find it presently; it requires an hour or more for the sugar to appear in the urine.

"The urine then eliminates all these substances in a manner more or less rapid. The prussiate of potash appears first, and the glucose last.

"There is another secretion in which the presence of sugar can be found; this is the gastric. The passage of the sugar into the stomach has surprised most of the observers who have seen long since that when diabetics vomited, although they had eaten nothing but flesh, the vomited matters were saccharine. When it was believed that diabetes proceeded from a perversion of the digestive functions, it was considered that the flesh was changed into sugar in the stomach. But one need not now be mistaken; the flesh is not saccharine. Bernard himself has observed that, in diabetics who vomit fasting, in the vomited matters the presence of sugar could be found. But this has only occurred when the disease is at its greatest intensity; and in all those cases, even in the animals which have been rendered artificially diabetic, it is much more difficult to obtain the passage of glucose iuto the gastric juice than into the urine.

"The sugar is formed, as we have seen, at the expense of the albuminous substances. In the healthy man it is clear that a part only of these matters is consumed for this purpose. The diabetic who forms much sugar expends a very large quantity of azotized material; the blood is impoverished; and, although the patient eats enormously, he gets thin like a man badly nourished. The liver takes in a manner the ration of the other organs, which undergo a considerable attenuation, because the albuminous elements are transformed into sugar.

"M. Bouchardat has prescribed the use of amylaceous and saccharine matters in the food of diabetics. The facts which Bernard has himself witnessed in the practice of M. Rayer prove clearly the utility of azotized aliment. In the regimen of these patients, writes Bernard, vegetable aliments ought to be forbidden, as it is evident they augment the functional activity of the liver. You know, also, that they are excitants of the kidneys; that they are much more diuretic than animal matters. Thus all the herbivora pass much more urine than carniverous animals. In the azotized regimen, diabetics have the advantage of food which is not diuretic.

"I have at great length reminded you of Mr. Bernard's views regarding the formation of sugar in the animal economy. As some of them are of so novel a character, and so little in accordance with the notions formerly held, I have thought it advisable to mention the experiments upon which he founds his opinions. That they will, upon further investigation, be more or less modified, is not improbable: but they have been very generally received by the most distinguished physiologists and pathologists.

"From M. Bernard's investigations, we learn the following facts of importance in reference to the saccharine plan of treating diabetes:—

"1. Sugar may be rationally administered to diabetic patients, inasmuch as the sugar found in the general circulation is almost always secreted by the liver, and as sugar introduced into the intestinal tube in its passage through the liver is there altered and converted into an emulsive substance, which serves to fatten these patients, and thus to counteract their tendency to emaciate.

"2. Substances which contain glucose—such as honey and fruits, should be given to diabetics in preference to those containing cane-sugar, because the latter is not destroyed when injected into the blood, but is constantly eliminated by the kidneys; whereas glucose can be destroyed in certain proportions.

"3. Cane-sugar would be beneficial to a certain extent; as, when taken into the intestine, it is in part, at least, transformed into glucose; but if given in too large proportions to be thus constantly transformed, the disease would be probably aggravated by the presence in the blood, and subsequent excretion by the kidneys, of the former variety of sugar.

"4. The glucose should be given in moderate quantities at a time, and frequently, rather than in large quantities at long intervals; because, when much sugar is taken fasting, it is absorbed too quickly to admit of its complete destruction in the liver, and it passes into the general circulation, whence it is eliminated in the urine."—*British Medical Journal*, May 8, 1858, from *The American Journal of the Medical Sciences.*

